# Development of microRNA-Based Glioblastoma Biomarkers Using Blood Plasma Specimens

**DOI:** 10.3390/diagnostics16050791

**Published:** 2026-03-06

**Authors:** Sophia Giliberto, Kenny K. Ablordeppey, Jacob Goldman, Melinda Yin, Rahul Chowdhury, Jacob Till, Kira Sheinerman, Sydney D. Finkelstein, Samuil Umansky, Alidad Mireskandari, Gyanendra Kumar, Erica L. Carpenter, Stephen J. Bagley

**Affiliations:** 1 Perelman School of Medicine, University of Pennsylvania, Philadelphia, PA 19104, USA; sophia.giliberto@pennmedicine.upenn.edu (S.G.); melinda.yin@pennmedicine.upenn.edu (M.Y.); rahul.chowdhury@pennmedicine.upenn.edu (R.C.); jacobetill@gmail.com (J.T.); erical@upenn.edu (E.L.C.); 2 DiamiR Biosciences Laboratory, 2 Church Street South, Suite B05, New Haven, CT 06519, USA; kablordeppey@diamirbio.com (K.K.A.); jgoldman@diamirbio.com (J.G.); ksheinerman@diamirbio.com (K.S.); sfinkelstein@diamirbio.com (S.D.F.); sumanski@diamirbio.com (S.U.); amireskandari@diamirbio.com (A.M.); gkumar@diamirbio.com (G.K.)

**Keywords:** microRNA, glioblastoma, liquid biopsy, biomarker

## Abstract

**Background:** Noninvasive biomarkers for the detection and monitoring of glioblastoma (GBM) are needed to improve clinical outcomes for patients. The objective of this pilot study was to evaluate the expression of a panel of 48 pre-selected microRNAs (miRNAs) in plasma specimens from GBM patients versus healthy controls to identify candidate miRNA biomarkers for noninvasive diagnosis of GBM. **Methods:** Selection of candidate miRNA biomarkers was based on a comprehensive literature review and data mining. RNA was extracted from plasma samples obtained prior to resection from patients with GBM (*n* = 30) and age- and sex-matched healthy controls (*n* = 30), as well as from matched FFPE GBM tissue samples when available (*n* = 3). Expression levels of 48 miRNAs were assessed in all samples, and expression data was processed using proprietary software to generate potential biomarkers and train linear classifiers. **Results:** Overall miRNA expression patterns were similar between matched plasma and FFPE tumor tissues in patients with GBM. miRNA levels were examined in pairs to determine the ratio between two miRNAs, which served to normalize the data. The top five miRNA pairs for distinguishing between GBM and healthy control plasma included miR-17-5p/miR-19b-3p (AUC 0.93, 95% CI = 0.870, 0.970), miR-20a-5p/miR-19b-3p (AUC 0.93, 95% CI = 0.870, 0.970), miR-93-5p/miR-92a-3p (AUC 0.92, 95% CI = 0.875, 0.965), miR-17-5p/miR-92a-3p (AUC 0.91, 95% CI = 0.865, 0.955), and miR-93-5p/miR-19b-3p (AUC 0.90, 95% CI = 0.850, 0.950). For the development of a multi-biomarker combination classifier consisting of up to three miRNA pair biomarkers, miRNA pairs with an AUC ≥ 0.8 were selected to build equal-weight linear classifiers. All possible combinations of three high-performing miRNA pairs were tested across the 60 samples. The top classifier (miR-20a-5p/miR-451a, miR-582-5p/miR-222-3p, and miR-17-5p/miR-222-3p) achieved an AUC value of 0.992, sensitivity of 0.93, specificity of 1, and accuracy of 0.97. **Conclusions:** These findings support the continued development of a plasma-based miRNA molecular diagnostic approach for the detection of GBM. The strong discriminatory performance observed in this study, including high AUC values, highlights the potential of circulating miRNA signatures as a minimally invasive diagnostic tool. As a pilot analysis, this work establishes a foundation for future prospective studies in larger, independent cohorts—including relevant disease control populations—to further define clinical performance, specificity, and utility in diagnostic and monitoring settings. Collectively, these results represent an important step toward the translation of plasma-based miRNA profiling into clinical application for GBM.

## 1. Introduction

Glioblastoma (GBM) is the most common primary brain malignancy in adults [1]. According to the World Health Organization (WHO) 2021 classification system, GBM should be diagnosed in the setting of an isocitrate dehydrogenase (*IDH*) wild-type diffuse and astrocytic glioma in adults if there is microvascular proliferation or necrosis or any of the following molecular alterations: *TERT* promoter mutation, *EGFR* amplification, or +7/−10 copy number changes [2]. Despite recent advances in the understanding of its biology and an aggressive standard of care consisting of chemoradiotherapy [3,4], GBM remains incurable and carries a 5-year survival rate of only 5–10% [1].

One of the major barriers to improving clinical outcomes in GBM has been the lack of non-invasive biomarkers for diagnosis and monitoring of the disease. On the diagnostic side, a significant challenge is accurate discrimination of GBM from other pathologies when an intracranial lesion is first identified on imaging. Current practice necessitates invasive neurosurgery to obtain tissue for diagnosis, which introduces risk and neurological morbidity. Reliable noninvasive strategies to diagnose GBM would improve patient care, either by providing diagnostic information preoperatively that can improve neurosurgical planning or by avoiding the need for highly invasive procedures altogether [5]. In terms of monitoring, standard MRI lacks the sensitivity to differentiate true progression from treatment-related changes, referred to as “pseudoprogression” [6,7,8], in patients with GBM who have received radiotherapy and other treatments. Given these challenges, liquid biopsy has emerged as a promising tool for diagnosis and monitoring of GBM [9].

Plasma isolated from peripherally drawn blood can be readily accessed and used as a biofluid for liquid biopsy [10]. While significant efforts have aimed to apply plasma cell-free DNA (cfDNA) assays to GBM [9,11,12], these tests have generally not performed well enough yet to warrant routine clinical use [13,14] or require expensive, time-consuming whole genome sequencing (WGS) [15]. An alternative liquid biopsy analyte that has been relatively understudied in GBM is microRNA (miRNA). miRNAs are 19–23 nucleotide long non-coding RNA molecules that exert post-transcriptional regulatory effects on many genes [16]. In the development of tumors, including GBM, they can act like oncogenes (oncomiRs) or tumor suppressor genes [17]. Recent mechanistic studies have elucidated the role of specific miRNA regulatory networks in glioblastoma pathogenesis, including their involvement in cell proliferation, invasion, angiogenesis, and immune evasion [18,19,20,21,22]. These insights provide a biological rationale for investigating circulating miRNA signatures as diagnostic biomarkers. Importantly, miRNAs circulate and are highly stable in plasma and have recently emerged as dynamic epigenetic biomarkers that can cost-effectively and easily be multiplexed for large-scale analytics [18,19]. Moreover, the organ-enriched nature of miRNAs makes them ideal targets for diagnostic test development [20].

We previously reported 37 brain-enriched miRNAs detected in plasma that could differentiate patients with clinical diagnoses of Alzheimer’s disease (AD), frontotemporal dementia (FTD), Parkinson’s disease (PD), and amyotrophic lateral sclerosis (ALS) from controls (AUCs > 0.9) and each other (AUCs from 0.77 to 0.93) [21]. We have also established and analytically validated a novel panel of 24 miRNAs detectable in plasma and enriched in AD-affected brain regions as a biomarker of mild cognitive impairment (MCI) and AD [23]. Recent studies have begun leveraging a similar approach to distinguish patients with GBM but have mostly identified individual miRNAs dysregulated in GBM patient plasma, notably miRNA-21 [24,25,26,27]. Few studies have developed multi-miRNA panels for GBM detection and monitoring, a more rigorous approach given the high pleiotropy of miRNAs. One prior report proposed a GBM expression profile composed of 35 miRNAs, though this was tissue-based [28]. Another study developed a blood-based miRNA signature using machine learning, which discriminated GBM patients from healthy controls with high accuracy, sensitivity, and specificity (81%, 79%, and 83%, respectively) [29]. However, neither of these studies reported *IDH*-mutant status, making it unclear whether participants fulfilled WHO criteria for GBM [2].

Here we constructed a panel of 48 GBM-relevant miRNAs based on an extensive literature review. Bridging prior work, we compared miRNA brain tissue expression and detectability in plasma to establish patterns between the two profiles [20,21]. Using their plasma miRNA expression profile, we sought to distinguish between plasma samples from healthy controls and GBM patients.

## 2. Materials and Methods

### 2.1. Patient Population and Study Design

Adult patients with newly diagnosed, histopathologically and/or molecularly confirmed glioblastoma at the University of Pennsylvania were enrolled in this pilot study between 1 January 2019, and 19 April 2024. Potential subjects with suspicion for a new diagnosis of high-grade glioma on MRI were approached for written informed consent before biopsy or surgical resection. Healthy control subjects with no history of cancer were enrolled under a separate biobanking protocol and were age and sex-matched to the GBM cohort. Blood samples from these patients were collected before routine endoscopy and colonoscopy procedures. Patients with a concurrent cancer and those enrolled in a clinical trial or receiving experimental therapy were excluded from the study. The study was approved by the University of Pennsylvania Institutional Review Board and conducted in accordance with the Declaration of Helsinki.

### 2.2. Plasma Collection and Processing

Preoperative plasma samples were collected from patients in the operating room immediately before resection. Whole blood samples were collected in either K_2_EDTA (Becton Dickinson, Franklin Lakes, NJ, USA) or Streck Cell-Free DNA blood collection tubes (Streck, La Vista, NE, USA). K_2_EDTA samples were stored and processed at 4 °C within two hours of collection by centrifuging at 1900× *g* for 10 min, followed by 3000× *g* for 15 min with the brake on at 4 °C. Streck samples were stored and processed at room temperature within five days of collection by centrifuging at 1600× *g* for 10 min, followed by 4122× *g* for 15 min with the brake off at 23 °C. One mL plasma aliquots were stored at −80 °C until use.

### 2.3. Tissue Collection and Processing

For three GBM subjects, formalin-fixed paraffin embedded sections were available for the resected tumor specimens. Ten serial, 4 micron sections were cut from the tissue block and mounted onto glass slides. Sections 1 and 10 were stained with hematoxylin and eosin; all others were unstained for nucleic acid extraction.

### 2.4. Development of the 48-miRNA Glioblastoma Panel for the Expression Analysis

The targeted selection of miRNA biomarkers for this pilot study was based on a comprehensive review of peer-reviewed literature and existing data on miRNAs linked to glioblastoma and to brain health [30,31,32,33,34,35,36,37], including their enrichment in the brain, association with inflammation, and involvement in GBM and general cancer-associated pathways. A total of 61 candidate miRNAs were initially selected, including those that are enriched in the brain, as well as those known to have tumor-suppressive roles or be involved in inflammation. These candidate miRNAs were analyzed in blood plasma samples from both GBM and healthy controls using RT-qPCR. Thirteen miRNAs were eliminated based on their poor detectability, or significant variability, or poor QC (Table 1).

### 2.5. RNA Isolation from Plasma Specimens

RNA was extracted from clinical plasma samples using the MagMAX mirVana Total RNA Isolation Kit (Thermo Fisher Scientific, Waltham, MA, USA). Briefly, 200 µL of plasma was incubated with 10 µL of proteinase K in 90 µL of proteinase K buffer for 30 min at 65 °C, followed by the addition of 200 µL lysis-binding mix and 40 µL RNA-binding magnetic beads. Next, 540 µL of isopropanol was added, and the samples were mixed and shaken for 15 min. RNA-bound beads were then washed with 300 µL wash solution I and 300 µL wash solution II using a magnetic stand. Beads were treated with 100 µL Turbo DNase, followed by the addition of 100 µL rebinding buffer and 200 µL isopropanol to rebind and precipitate RNA. After additional washes to remove contaminants, RNA was eluted from the beads with 100 µL of kit-provided elution buffer at 65 °C and stored at −70 °C. According to DiamiR’s standard protocol, prior to RT-qPCR, 100 µL of eluted RNA was diluted with 225 µL nuclease-free water to yield a final volume of 325 µL. This diluted RNA preparation was used for all subsequent assays.

### 2.6. RNA Isolation from FFPE Specimens

GBM tumors were surgically resected from three patients and were fixed in formalin and embedded in parafilm (FFPE). Tumor-containing regions of the FFPE tissues were identified by a pathologist and carefully dissected and processed for RNA extraction using ThermoFisher’s RecoverAll™ Total Nucleic Acid Isolation Kit for FFPE samples. FFPE tissue slides were deparaffinized by sequential immersion in Envirene baths and a 70% ethanol bath, followed by air drying to remove residual wax. The tissue was then scraped from the slides and transferred into tubes containing digestion buffer from the RNA isolation kit. RNA extraction was performed on the tumor sections from all three samples. Proteinase K was added to the tissue buffer mixture, and the samples were incubated overnight at 55 °C. The following day, isolation additive and 100% ethanol were introduced, and the lysates were passed through filter cartridges via centrifugation. Flow-through was discarded, and the cartridges were washed with Wash I and Wash 2/3 solutions. DNAse treatment was performed, followed by an additional round of Wash I and Wash 2/3. Finally, RNA was eluted in 60 µL of preheated nuclease-free water. The purified RNA was subsequently used for RT-qPCR to evaluate miRNA expression levels in the tumor tissues.

### 2.7. MicroRNA Expression Analysis Using LNA-Based Qiagen RT-qPCR Technology

Before the reverse transcription (RT) step, a bulk preparation of synthetic spike-in RNA (UniSp6) was made according to the manufacturer’s instructions (miRCURY LNA RNA Spike-in Kit, Qiagen, Hilden, Germany). For RT, 20 µL of master mix containing 6 µL nuclease-free water, 8 µL 5 × miRCURY RT reaction buffer, 4 µL 10 × miRCURY RT enzyme mix, and 2 µL synthetic spike-in RNA was combined with 20 µL of isolated RNA in designated wells of a 96-well plate. The RT reaction was carried out at 42 °C for 60 min, followed by enzyme inactivation at 95 °C for 5 min, after which the plate was held at 4 °C. This sequence-independent RT generated cDNA corresponding to all targeted miRNAs in each well. The resulting cDNA was amplified using Qiagen’s Locked Nucleic Acid (LNA) RT-qPCR technology for the 48 miRNAs included in the study panel. Sample-specific, primer-deficient master mixes were prepared such that each 10 µL reaction contained 3.7 µL nuclease-free water, 0.20 µL ROX reference dye, 5 µL 2× miRCURY LNA SYBR Green Master Mix, and 1.07 µL RT product. Each 10 µL master mix was dispensed into the designated wells of the miRCURY LNA miRNA Custom PCR Panel in a 384-well plate. The custom panel enabled simultaneous testing of four samples across 48 miRNAs, along with recommended controls (BlankSpot, UniSp3, and UniSp6). Each RT-qPCR run included both positive and negative controls. The positive control for all 48 miRNAs was prepared from synthetic RNA oligonucleotides (Integrated DNA Technologies, Coralville, IA, USA), while no-template controls (NTC) served as negative controls. RT-qPCR was performed on a QuantStudio 7 Flex instrument (Thermo Fisher Scientific Inc., Waltham, MA, USA) using the following program: initial heat activation at 95 °C for 2 min (1 cycle), followed by 40 cycles of denaturation at 95 °C for 10 s and annealing/extension at 60 °C for 56 s. A melt-curve analysis was then performed from 60 to 95 °C. The raw Ct values for all assays were exported for subsequent data analysis.

### 2.8. Analysis of miRNA Expression in Glioblastoma FFPE Tissues Compared to Matched Plasma

The tumor cells within FFPE tissue sections were micro-dissected for RNA extraction as described above. The extracted RNA was analyzed by qPCR using the 48-miRNA panel. In parallel, RNA was extracted from the corresponding plasma specimens of the same patients and subjected to the same qPCR analysis. The Excel software 2010 program was used for the analysis of the raw Ct data of the 48 miRNA expression levels in the matched plasma specimens and FFPE tissues of three patients.

### 2.9. MicroRNA Expression Analysis and Classifier Development

Expression levels (Ct data) of 48 miRNAs from the GBM panel were measured in plasma samples from the GBM patients and the age- and sex-matched healthy controls to assess whether the two groups can be distinguished using miRNA pair classifiers. Expression data was processed using DiamiR proprietary software (version 16.1.174.FULL) to generate potential biomarkers and train linear classifiers. The Ct data was normalized by forming all possible pairwise ratios of the 48 miRNAs, resulting in 1128 candidate biomarkers [21,154,155]. Each pair was evaluated for sensitivity, specificity, accuracy, and AUC when used to differentiate GBM samples from controls. Pairs achieving an AUC ≥ 0.8 advanced to the next stage of analysis. In the next phase, miRNA pairs were combined to form equal-weight classifiers, in which each pair ratio contributed equally to the model. For each classifier, the probability of a sample being classified as GBM was modeled as a linear combination of the selected pair ratios. All possible combinations of three high-performing miRNA pairs were tested. Each classifier was applied to all samples, and sensitivity, specificity, accuracy, and AUC were recorded to assess classification performance.

For pair ratio calculation, all possible pairwise ratios of the 48 miRNAs were calculated, resulting in 1128 candidate biomarkers (48 × 47/2 = 1128 unique pairs). For each pair, the ratio was computed as: Ratio = 2^(Ct miRNA1 − Ct miRNA2). This ratio-based approach serves as an internal normalization strategy that reduces technical variability by canceling out systematic effects that similarly impact both miRNAs in the pair [20,21].

For feature selection and single-pair classifier evaluation, each miRNA pair was evaluated as a binary classifier using logistic regression. For each pair, sensitivity, specificity, accuracy, and area under the receiver operating characteristic curve (AUC) were calculated when used to differentiate GBM samples from controls. Pairs achieving AUC ≥ 0.8 were selected for inclusion in multi-biomarker classifiers. This threshold was chosen a priori based on previously published guidelines for biomarker development, indicating that AUC ≥ 0.8 represents good discriminatory performance.

For multi-biomarker classifier development, the 76 miRNA pairs achieving AUC ≥ 0.8 were used to construct multi-biomarker classifiers consisting of three miRNA pairs. Multi-biomarker classifiers were built as equal-weight linear combinations of miRNA pair ratios. Specifically, the probability P of a sample originating from a GBM patient was modeled as: P(GBM) = 1/(1 + e^(−score)), where score = (Ratio_1_ + Ratio_2_ + Ratio_3_)/3, with equal weights assigned to each pair. All possible combinations of three pairs from the 76 high-performing pairs were systematically evaluated. Classification thresholds were set at *p* = 0.5 (i.e., samples with *p* ≥ 0.5 classified as GBM; samples with *p* < 0.5 classified as control).

Model selection and performance evaluation were conducted on the same dataset (all 60 samples). This approach is appropriate for exploratory biomarker discovery in a pilot study but may result in overfitting and optimistically biased performance estimates. Independent validation in separate cohorts is essential to determine the true predictive accuracy of these classifiers. We evaluated 1128 possible miRNA pairs as candidate biomarkers. No correction for multiple testing (e.g., Bonferroni or false discovery rate adjustment) was applied during the exploratory biomarker discovery phase, as this was a hypothesis-generating pilot study, and we acknowledge that this may increase the risk of identifying false-positive associations. The AUC threshold of ≥0.8 was used to prioritize promising candidates for further evaluation, but independent validation will be essential to confirm which associations represent true biological signal versus statistical noise.

## 3. Results

### 3.1. Patient Characteristics

Thirty patients with GBM and 30 age and sex-matched healthy study participants were enrolled. Patient characteristics are displayed in Table 2.

### 3.2. Analysis of miRNA Expression in Glioblastoma FFPE Tissues Compared to Matched Plasma

**Figure 1:**
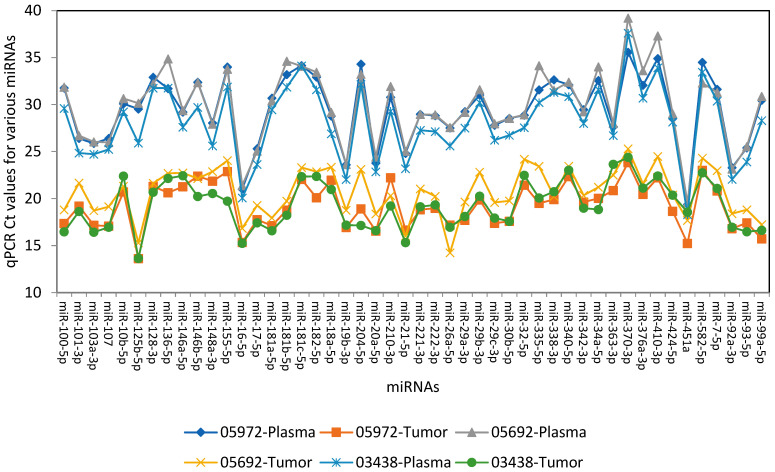
48 miRNA expression levels in plasma and FFPE tissues. qPCR Ct values are displayed (Y-axis; higher Ct indicates lower expression) for the 48 miRNAs included in the panel (X-axis).

Quantitative PCR-based miRNA expression analysis was carried out on the miRNAs isolated from the tumor sections of FFPE slides from three patients, concurrent with the miRNAs isolated from the plasma of the same 3 patients as described in Section 2. The graph representing miRNA expression levels in these specimens is shown in Figure 1. The resulting graph displays qPCR Ct values (Y-axis; higher Ct indicates lower expression) for the 48 miRNAs included in the panel (X-axis). The data demonstrate that (i) as expected, miRNA expression levels were generally higher in tumor tissues than in plasma, although all 48 miRNAs were detectable in both sample types, and (ii) the overall expression patterns of these miRNAs were similar between plasma and FFPE tumor tissues. These findings support the potential utility of plasma miRNA expression as a surrogate for glioblastoma tumor tissue profiling.

### 3.3. MicroRNA Expression Analysis and Classifier Development Using GBM and Control Cohorts

MicroRNA expression analysis for the identification of miRNA pair biomarkers for the classifier development was carried out using the RNA samples isolated from the plasma specimen collected before surgery from the 30 patients with GBM, along with age- and sex-matched samples from 30 non-GBM controls. Total RNA was extracted and analyzed for the expression of a 48-miRNA panel. Pairwise miRNA-based classifiers were then trained, as described in Section 2 under classifier development, to distinguish GBM from control samples. Using DiamiR’s proprietary software, more than 70 miRNA pairs resulted in an AUC of ≥0.8. Table 3 presents the top five pairs, which included miR-17-5p/miR-19b-3p (AUC 0.93), miR-20a-5p/miR-19b-3p (AUC 0.93), miR-93-5p/miR-92a-3p (AUC 0.93), miR-17-5p/miR-92a-3p (AUC 0.91), and miR-93-5p/miR-19b-3p (AUC 0.90).

**Figure 2:**
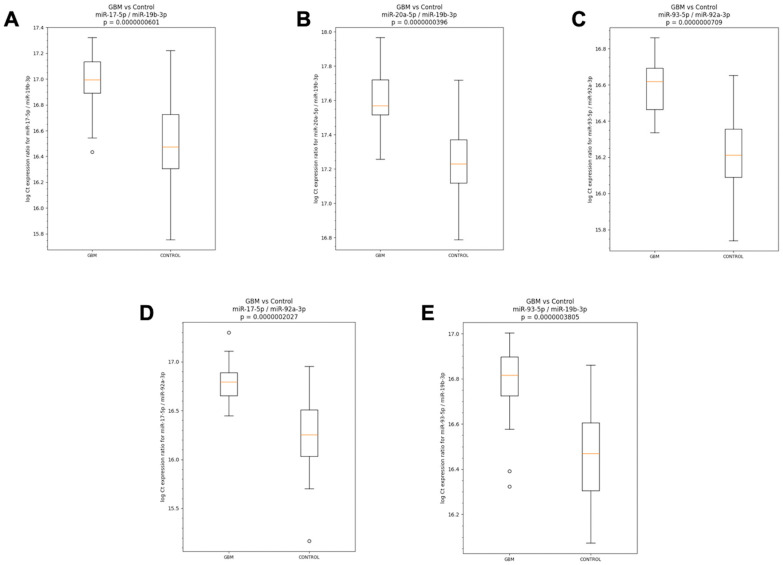
(**A**–**E**) Boxplots showing the spread of miRNA expression ratios for 5 miRNA pair biomarkers in GBM and control cohorts. Each subfigure corresponds to an miRNA pair, with boxplots showing the minimum, first quartile, median (orange line), third quartile, and maximum expression ratio values in log scale for the GBM and control cohorts. The *p*-value for a Mann–Whitney U Test is reported above each subfigure.

To visualize the miRNA spread of these five miRNA pair biomarkers between GBM and control cohorts, the qPCR data used for the identification of these biomarkers was utilized to generate standard box-and-whisker plots shown below in Figure 2. Each pair shows a visible difference between the two groups (Mann–Whitney U Test), corroborating their strong performance metrics for classification.

### 3.4. Development of a Multi-Biomarker Combination Classifier for GBM vs. Control Cohorts

For the development of a multi-biomarker combination classifier consisting of up to three miRNA pair biomarkers, with much higher specificity, sensitivity and accuracy for cohort segregation, 76 miRNA pairs with an AUC ≥ 0.8 (Table 3) were selected to build equal-weight linear classifiers using the DiamiR software, which modeled the probability of a sample originating from a glioblastoma patient as a linear combination of pair ratio values. All possible combinations of three high-performing miRNA pairs were tested across 60 samples, and sensitivity, specificity, accuracy, and AUC were recorded. Six classifiers met the predefined criteria (Table 4). Notably, Classifier #1 (miR-20a-5p/miR-451a, miR-582-5p/miR-222-3p, miR-17-5p/miR-222-3p) with the highest AUC value of 0.992, achieved a sensitivity of 0.93, specificity of 1, and accuracy of 0.97.

**Figure 3:**
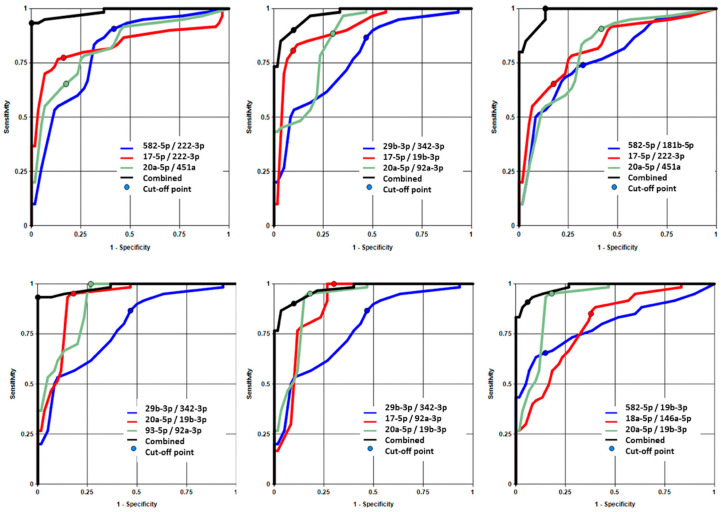
Receiver Operating Characteristic (ROC) curve from the combination of three miRNA pair biomarkers.

To visualize the comparative performance of individual miRNA pair biomarkers and combinations of multiple pair biomarkers, the ROC curves for the respective classifiers shown in Table 4 were generated with one set of axes for each of the top three performing combined classifiers (Figure 3). Each subfigure shows four ROC curves: one for each miRNA pair biomarker in the combined group of three showing the performance of that marker’s individual classifier and one showing the performance of the combined classifier as reported in Table 4. This highlights the performance benefits of combining multiple biomarkers into a single classifier signature.

## 4. Discussion

Noninvasive biomarkers for diagnosis and monitoring of GBM remain a major unmet need in neuro-oncology. In current practice, neuroimaging cannot reliably distinguish GBM from other intracranial neoplasms, and a confirmed diagnosis requires tissue acquisition via a brain biopsy or a craniotomy for tumor resection [156]. In addition, MRI scans are unable to accurately discriminate between true tumor progression and pseudoprogression in patients with GBM who have received prior radiotherapy or immunotherapy [157]. As a result, invasive brain tissue sampling is often required to understand the status of a patient’s tumor in the setting of equivocal radiographic changes. Here we conducted a pilot study to explore the potential of circulating miRNAs detectable in blood plasma as a non-invasive blood test for detection of GBM. We found that a set of 48 GBM-relevant miRNAs are expressed similarly in matched tumor tissue and plasma specimens in patients with GBM. We were also able to reliably distinguish GBM patient plasma samples from those obtained from healthy controls based on expression of plasma miRNA pairs and plasma miRNA expression profiles, with even higher AUCs approaching 1.0 when using a multi-biomarker combination classifier consisting of three miRNA pairs. Taken together, these data suggest a potential role for a plasma multi-miRNA panel for noninvasive detection and monitoring of GBM.

While the AUC values achieved by our classifiers (up to 0.992) are statistically impressive, it is important to contextualize these findings within the clinical decision-making framework for patients with suspected brain tumors. The true clinical utility of these biomarkers depends not solely on their ability to distinguish GBM patients from healthy controls, but rather on their performance in clinically relevant scenarios including: (1) pre-operative risk stratification when an intracranial mass is detected on imaging; (2) differentiation of GBM from other high-grade gliomas, brain metastases, and non-neoplastic lesions; (3) detection of minimal residual disease post-surgery; and (4) distinction between true tumor progression and treatment-related changes (pseudoprogression).

The present study was designed to evaluate biomarker performance in a well-defined cohort of newly diagnosed, treatment-naive GBM patients compared with neurologically healthy controls. This design allowed for a clear assessment of the discriminatory miRNA signal under controlled conditions. Clinical decision-making regarding surgical biopsy or treatment modification, however, extends beyond measures of sensitivity and specificity and requires integration of procedural risk, diagnostic uncertainty, and potential consequences of delayed intervention. Accordingly, future studies will focus on evaluating the utility of these biomarkers in clinically complex and heterogeneous populations to determine their capacity to inform management decisions and improve patient outcomes. Establishing clinical utility in real-world settings represents an important next step in translating these findings into clinical practice.

The current standard of care for suspected GBM relies on MRI findings (including contrast enhancement, necrosis, mass effect, and diffusion characteristics) combined with invasive tissue sampling. Advanced MRI techniques (perfusion imaging, MR spectroscopy, diffusion tensor imaging) can suggest high-grade glioma but cannot definitively establish a diagnosis of GBM or distinguish progressive/recurrent GBM from treatment effect. Our miRNA biomarkers cannot replace tissue diagnosis for establishing WHO classification and molecular status of gliomas (IDH mutation, MGMT methylation, 1p/19q codeletion), which remain essential for treatment planning. Rather, the potential value of plasma miRNA biomarkers lies in: (1) Pre-surgical risk assessment to guide surgical planning and patient counseling; (2) Non-invasive monitoring for disease recurrence when repeat tissue sampling is not feasible or safe; and (3) Early detection of recurrence when imaging findings are equivocal. To truly demonstrate incremental value, future studies must compare the diagnostic accuracy of miRNA biomarkers + standard imaging versus imaging alone, using appropriate metrics such as net reclassification improvement (NRI) and integrated discrimination improvement (IDI). These analyses would assess whether adding miRNA testing meaningfully improves patient classification beyond existing tools.

Other plasma-based liquid biopsy approaches have been evaluated for GBM with mixed success. Although targeted next generation sequencing (NGS) assays using cfDNA have not been sensitive enough for routine clinical use [11,12,158], alternative cfDNA approaches have recently demonstrated more encouraging results. Ali et al. leveraged tumor-specific amplified DNA junctions in patients with high-grade glioma to develop personalized polymerase chain reaction (PCR) assays [159]. Early data using this approach in five patients demonstrated potential for longitudinal monitoring, although larger studies are needed. Using machine learning methods, Mathios and colleagues developed a genome-wide cfDNA fragmentation method for the detection of glioma in patient plasma samples [15]. The investigators detected brain cancer across all-grade gliomas with an AUC of 0.90 and validated these results in an independent prospectively collected cohort. Nevertheless, this approach utilizes WGS and is thus unlikely to be scalable for clinical use in its current form. Other novel approaches have included analysis of plasma cfDNA methylomes [5], as well as the use of focused ultrasound to transiently open the blood–brain barrier and enhance the yield of plasma cfDNA [160]. Compared to these cfDNA-based assays, miRNA-based liquid biopsies have several potential advantages for clinical diagnosis, treatment monitoring, and tumor characterization. Circulating miRNAs, released extracellularly for local and remote activity, are highly stable and more abundant than cell-free DNA [161], which is often scarce in glioblastoma plasma due to limited tumor shedding [12]. Further, circulating miRNAs show far greater diversity, thereby capable of manifesting intrinsic differences in glioma biology, including clinical aggressiveness [162]. Brain-enriched miRNAs are especially useful in this regard, providing the means to readily evaluate the status of gliomas from outside the central nervous system [21,22]. Most importantly, by using a panel of miRNA analytes, as described in this report, both tumor growth promotion as well as growth suppression can be analyzed together with host response with respect to inflammation and immune modulation [163].

Other miRNA approaches for the detection of GBM have been reported previously. Rezaie et al. highlight the role of miRNAs in the pathogenesis of glioblastoma by providing a summary of studies that reported dysregulation of these epigenetic effectors in this kind of brain cancer [22]. Regazzo et al. developed a serum miRNA signature that could distinguish GBM from lower-grade gliomas [164]. In this study of 30 patients with various grades of glioma, serum miRNA expression correlated with paired tissue sample expression for two miRNAs. The authors also observed AUC values ranging from 0.75 to 0.87 for distinguishing GBM from lower-grade gliomas. In another study, Bustos and colleagues used an miRNA whole transcriptome assay to compare plasma samples from patients with GBM to control samples from normal healthy donors [165]. 265 miRNAs were found differentially expressed in GBM patients (*n* = 45) compared to healthy controls (*n* = 73), and two miRNAs (miR-3180-3p and miR-5739) were found to have particularly high specificity (87.7% and 79.5%, respectively) and sensitivity (100% and 92.3%, respectively). The authors also demonstrated a significant positive correlation between detection of miRNAs in plasma and matched GBM tissue samples. More recently, Ali et al. evaluated three specific circulating miRNAs (miR-29a, miR-106a, and miR-200a) and established an association with prognosis in patients with GBM [166]. Although the sample size of only 25 GBM patients was limited for the evaluation of survival outcomes, the investigators reported that low expression levels of miR-106a were associated with better PFS and OS.

Our results compare favorably to the aforementioned studies. After training logistic regression classifiers to distinguish GBM patients from healthy controls, we found that five pairs of miRNAs had AUC values greater than 0.90, with the top pair being miR-17-5p/miR-19b-3p (AUC 0.93). miR-17-5p is a well-studied oncogenic miRNA with roles in promoting tumor cell proliferation, cell-cycle progression, angiogenesis, and immune modulation [154]. In GBM, miR-17-5p is consistently upregulated, promotes stemness, invasion, and therapeutic resistance, and is associated with worse prognosis [35,155]. Similarly, miR-19b-3p is also a potent oncogenic miRNA that is known to silence tumor suppressor genes and has important roles in GBM, including activation of the JAK-STAT pathway and reduction in PTEN levels [47,52]. When we evaluated a multi-biomarker classifier consisting of up to three miRNA pair biomarkers, much higher specificity, sensitivity and accuracy for distinguishing GBM from controls were obtained. miRNA-17-5p was also a part of the highest performing classifier, which included microRNAs miR-20a-5p/miR-451a, miR-582-5p/miR-222-3p, and miR-17-5p/miR-222-3p and achieved an AUC value of 0.992 and an accuracy of 0.97. With accuracy this high, these results demonstrate the potential for a noninvasive miRNA-based molecular test to detect glioblastoma using plasma.

Overall, this study represents an initial evaluation of the utility of miRNAs as diagnostic markers for GBM and provides a strong foundation for continued investigation. This study was designed to establish biological signal and classifier feasibility within a well-characterized cohort consistent with development-phase biomarker studies. Model training and performance assessment were conducted within the same dataset to define discriminatory capacity under controlled conditions. Accordingly, the reported performance metrics should be interpreted within the framework of this developmental design.

Several aspects of this study provide direction for future investigations. The tissue–plasma concordance analysis, performed in three matched patients, demonstrated encouraging alignment in expression patterns and supports the biological relevance of circulating miRNA signatures versus tissue expression. However, expansion of this analysis in larger cohorts with additional matched tissue–plasma pairs will be necessary to increase confidence in our findings and further clarify the relationship between tumor-derived and circulating miRNA expression in GBM.

This exploratory analysis evaluated 1128 candidate miRNA pairs to comprehensively assess potential discriminatory signals. Although multiple testing correction was not applied in this hypothesis-generating phase, the approach enabled broad signal discovery and identification of high-performing candidates. Independent validation in external datasets will be an important next step to confirm reproducibility and distinguish robust biomarkers from statistical variability.

The cohort size (*n* = 30 per group) was appropriate for an initial translational evaluation and allowed detection of strong discriminatory performance. However, this study lacked an independent validation cohort, and larger studies will be needed to enable greater statistical precision and support subgroup analyses across clinically relevant variables such as tumor location, extent of resection, and molecular subtype. Importantly, there was a racial imbalance between the GBM cohort (93.3% White) compared to the control cohort (73.3% White, 23.3% Black/African American), which represents a potential confounding variable that will need to be addressed in future validation studies.

Finally, the use of neurologically healthy controls provided a clear initial framework for signal detection. Future studies incorporating disease control populations—including lower-grade gliomas, meningiomas, brain metastases, primary CNS lymphoma, and non-neoplastic conditions that may mimic GBM radiographically—will be important to further define diagnostic specificity and real-world clinical performance.

Collectively, these considerations outline a clear pathway for continued validation and clinical translation of plasma-based miRNA profiling in GBM [22]. The specificity of our miRNA biomarkers for GBM versus these alternative diagnoses remains unknown and represents a critical next step for validation. Future studies should include: (i) patients with IDH-mutant gliomas to assess discrimination between GBM and lower-grade tumors; (ii) patients with other primary brain tumors to evaluate specificity; (iii) patients with non-neoplastic intracranial pathology to reduce false positives; and (iv) serial monitoring in the same patients pre- and post-surgery to assess for treatment response and recurrence detection. Without such comparisons, the clinical utility of these biomarkers for differential diagnosis remains speculative.

In summary, our findings provide positive proof-of-concept support for the development of an miRNA plasma-based molecular diagnostic test for detecting GBM, with potential for both noninvasive diagnosis and, if demonstrated in subsequent studies, on-therapy monitoring. The high AUC values observed are encouraging but must be interpreted in context, given the absence of independent validation. Our data warrant larger, prospective studies to evaluate the clinical utility of miRNA-based plasma assays for discrimination of GBM from other intracranial tumors in patients with a brain mass detected on MRI, as well as for distinguishing pseudo-progression from true tumor progression following treatment of GBM. We also plan to evaluate the performance of miRNA-based plasma biomarkers in other tumors of the central nervous system, including IDH-mutant glioma. Most importantly, future validation studies must include disease controls, independent test cohorts, ethnically diverse populations, and a demonstration of incremental diagnostic value beyond standard imaging to establish whether these biomarkers can meaningfully improve clinical decision-making and patient outcomes.

## Figures and Tables

**Table 1:** Selected miRNAs.

miRNA	Role in GBM	Brain Enrichment	Target/Pathway	References
miR-7-5p	Tumor suppressor	+	SATB1	[38,39,40]
miR-10b-5p	OncomiR	+	Proliferation and invasion	[41,42,43]
miR-16-5p	Tumor suppressor	+	Cell cycle and apoptosis; Wip1-ATM-p53	[44,45,46]
miR-17-5p	Context dependent	+	PTEN; CXCL14	[44,47,48,49]
miR-18a-5p	OncomiR	−	CBX7; Neogenin	[50,51]
miR-19b-3p	OncomiR	+	JAK-STAT; PTEN	[44,52,53]
miR-20a-5p	OncomiR	+	LRIG1; CELF2	[54,55,56]
miR-21-5p	OncomiR	+	PDHA1	[54,57,58]
miR-26a-5p	OncomiR	+	CDK4; PTEN	[44,59,60]
miR-29a-3p	Context dependent	+	PTEN; PDGF; PI3K-AKT	[44,61,62,63]
miR-29b-3p	Tumor suppressor	+	BCL2L2; autophagy	[44,64,65]
miR-29c-3p	Tumor suppressor	+	MMP2; Gab1	[44,66,67]
miR-30b-5p	Context dependent	+	PRRT2; MTDH	[44,68,69]
miR-32-5p	Context dependent	−	PTEN; EZH2	[70,71]
miR-34a-5p	Tumor suppressor	−	MAPK	[38,72]
miR-92a-3p	OncomiR	+	CDH1/β-catenin; Notch-1/Akt; Bim	[73,74,75]
miR-93-5p	Context dependent	−	Inflammation related; PI3K/Akt; integrin-β8; MMP2	[76,77,78,79]
miR-99a-5p	Tumor suppressor	+	FGFR3-TACC3	[80,81,82]
miR-100-5p	Tumor suppressor	+	SMRT/NCOR2	[44,83,84]
miR-101-3p	Tumor suppressor	+	KLF6; EZH2; SOX9	[44,85,86,87]
miR-103a-3p	Tumor suppressor	+	BDNF	[44,88,89]
miR-107-3p	Tumor suppressor	+	Notch2; CDK6	[90,91,92,93]
miR-125b-5p	OncomiR	+	p53; p38MAPK; TNFAIP3; NKIRAS2	[43,94,95]
miR-128-3p	Tumor suppressor	+	c-Met/EMT; IL-8; Bmi-1	[54,96,97,98]
miR-136-5p	Tumor suppressor	+	BCL9L; AEG-1; Bcl-2	[54,99,100]
miR-146a-5p	Tumor suppressor	+	Inflammation related; Notch1; POU3F2/SMARCA5	[54,101,102,103]
miR-146b-5p	Tumor suppressor	−	EGFR; MMP16	[104,105]
miR-148a-3p	Context dependent	+	ERRFI1; MIG6; BIM; SIRT7; DNMT1	[106,107,108,109,110]
miR-155-5p	OncomiR	−	Inflammation related; ACOT12; PI3K/AKT	[111,112,113]
miR-181a-5p	Tumor suppressor	+	MAPK; FBXO11	[44,114,115]
miR-181b-5p	Tumor suppressor	+	SP1; Bcl-2	[44,116,117]
miR-181c-5p	Tumor suppressor	+	Inflammation related; wnt/β-catenin; TGF-β	[118,119,120,121]
miR-182-5p	OncomiR	+	KLF2; KLF4; STAT3	[122,123,124]
miR-204-5p	Tumor suppressor	+	SOX4; EphB2; RAB22A	[54,125,126]
miR-210-3p	OncomiR	+	HIF1α/HIF2α; TGF-β	[54,127,128]
miR-221-3p	OncomiR	+	HHIP; PUMA	[54,129,130]
miR-222-3p	OncomiR	+	PUMA; PTPμ	[54,130,131]
miR-335-5p	OncomiR	+	Daam1	[36,38,54]
miR-338-3p	Tumor suppressor	+	Proliferation and neuronal maturation; OXSM	[54,132,133]
miR-340-5p	Tumor suppressor	+	GTF2E2; Bcl-w; Sox2	[118,134,135]
miR-342-3p	Tumor suppressor	+	CDK6	[44,136,137]
miR-363-3p	OncomiR	−	CELF2; PDHB	[138,139]
miR-370-3p	Tumor suppressor	+	HMGA2; HIF1A; MGMT	[80,140,141]
miR-376a-3p	Tumor suppressor	+	KLF15; SP1	[54,142,143]
miR-410-3p	Tumor suppressor	+	STAT3; RAP1A	[39,54,144,145]
miR-424-5p	Tumor suppressor	+	AKT1; RAF1; KIF23	[146,147,148]
miR-451a-5p	Context dependent	+	LKB1/AMPK; PI3K/AKT	[54,149,150,151]
miR-582-5p	OncomiR	−	Caspase 3; Caspase 9; Bim	[152,153]

**Table 2:** Patient characteristics.

Patient	Healthy Controls	Glioblastoma	*p*-Value
	*n* = 30	*n* = 30	
**Age**			
Median (Min–Max)	64.70 (52.81–78.57)	67.90 (45.10–80.76)	0.2617 *
<65, *n* (%)	15 (50%)	11 (36.67%)	0.4348 **
>65, *n* (%)	15 (50%)	19 (63.33%)	
**Sex, *n* (%)**			
Female	11 (33.67%)	12 (40.00%)	>0.9999 **
Male	19 (63.33%)	18 (60.00%)	
**Race, *n* (%)**			
White	22 (73.33%)	28 (93.33%)	0.0034 **
Black or African American	7 (23.33%)	0 (0.00%)	
Other	1 (3.33%)	0 (0.00%)	
Unknown	0 (0.00%)	2 (6.66%)	

* Mann–Whitney Test, ** Fisher’s exact test.

**Table 3:** miRNA pair classifiers identified using GBM vs. control cohorts.

miRNA Pairs	Sensitivity	Specificity	Accuracy	AUC	95% CI	Mann–Whitney *p*-Value
miR-17-5p/miR-19b-3p	0.81	0.9	0.85	0.93	(0.890, 0.970)	$6.01\times 10^{-8}$
miR-20a-5p/miR-19b-3p	0.95	0.82	0.89	0.93	(0.890, 0.970)	$3.96\times 10^{-8}$
miR-93-5p/miR-92a-3p	1	0.73	0.87	0.92	(0.875, 0.965)	$7.09\times 10^{-8}$
miR-17-5p/miR-92a-3p	1	0.7	0.85	0.91	(0.865, 0.955)	$2.207\times 10^{-7}$
miR-93-5p/miR-19b-3p	0.81	0.81	0.81	0.9	(0.850, 0.950)	$3.805\times 10^{-7}$

**Table 4:** Combinations of three miRNA pairs with AUC > 0.8.

#	Pair 1	Pair 2	Pair 3	Sensitivity	Specificity	Accuracy	AUC
1	20a-5p/451a	582-5p/222-3p	17-5p/222-3p	0.93	1	0.97	0.992
2	29b-3p/342-3p	17-5p/19b-3p	20a-5p/92a-3p	0.97	0.97	0.97	0.990
3	582-5p/181b-5p	17-5p/222-3p	20a-5p/451a	1	0.87	0.93	0.990
4	29b-3p/342-3p	20a-5p/19b-3p	93-5p/92a-3p	0.93	1	0.97	0.989
5	29b-3p/342-3p	20a-5p/19b-3p	17-5p/92a-3p	0.97	0.97	0.97	0.989
6	582-5p/19b-3p	20a-5p/19b-3p	18a-5p/146a-5p	0.93	0.97	0.95	0.989

## Data Availability

The raw qPCR Ct values and normalized expression data generated in this study are available from the corresponding author upon reasonable request. Access to data will be provided in accordance with institutional data sharing policies and patient privacy regulations.
